# Nitrogen Retention in Headwater Streams: The Influence of Groundwater-Surface Water Exchange

**DOI:** 10.1100/tsw.2001.272

**Published:** 2001-11-09

**Authors:** S. A. Thomas, H. M. Valett, P.J.. Mulholland, C.S. Fellows, J.R. Webster, C.N. Dahm, C.G Peterson

**Affiliations:** Department of Biology, Virginia Polytechnic Institute, Blacksburg 24060, USA

**Keywords:** streams, hyporheic zone, nitrogen, spiraling, groundwater-surface water interaction

## Abstract

Groundwater-surface water (GW-SW) interaction lengthens hydraulic residence times, increases contact between solutes and biologically active surfaces, and often creates a gradient of redox conditions conducive to an array of biogeochemical processes. As such, the interaction of hydraulic patterns and biogeochemical activity is suspected to be an important determinant of elemental spiraling in streams. Hydrologic interactions may be particularly important in headwater streams, where the extent of the GW-SW mixing environment (i.e., hyporheic zone) is proportionately greater than in larger streams. From our current understanding of stream ecosystem function, we discuss nitrogen (N) spiraling, present a conceptual model of N retention in streams, and use both of these issues to generate specific research questions and testable hypotheses regarding N dynamics in streams.

## INTRODUCTION

Increased availability of fixed nitrogen (N) resulting from human activity has become one of the most conspicuous aspects of global change[1]. Industry now produces more biologically available N than is fixed by biotic processes in ecosystems worldwide[2]. N pollution is especially pronounced in urban areas, where deliberate (N fixation by legumes, food, and fuel import) and inadvertent (e.g., conversion of $N_2$ to nitrate-N [$NO_3$-N] during fossil fuel combustion) sources may account for >90% of N inputs[3,4]. Reviewing a 7-year data set that included over 300 surface water sites, Smith and Alexander[5] and Smith et al.[6] identified increasing $NO_3$-N, the most mobile form of inorganic N, as the most evident trend in water chemistry compared to changes in 22 other water quality variables. In large river systems, N loading to the oceans is correlated to human population density and has increased as much as 20-fold since preindustrial times[7,8].

The flood of fixed N within the biosphere has altered the chemistry of the atmosphere, soils, and waters. In turn, these changes have influenced ecological processes in environments ranging across forest, grassland, montane, marine, and freshwater ecosystems[9,10,11,12]. Elevated N availability should lead to a greening of the biosphere because N is an important macronutrient that frequently limits plant production in both terrestrial and aquatic environments. However, the distribution of anthropogenic N is heterogeneous, and regions receiving the highest rates of N loading often exhibit diminished ecological integrity. Several forests in North America, Europe, and China are in decline as a result of N saturation[13].

Mass balance studies for major watersheds within the U.S. indicate that ~60 to 80% of the N added to catchments is not exported to the oceans and thus is missing from budgetary accounts, presumably stored within these watersheds[8]. Even so, the 20 to 40% of added N that does leave the watershed represents a level of N loading to estuaries and coastal oceans that is unacceptable if large-scale oxygen depletion results, as has been documented in the Chesapeake Bay and Mississippi Delta[14,15]. Consequently, a better understanding of N cycling at all spatial scales is required if society is to successfully manage and protect sensitive ecosystems and important ecosystem services, such as drinking-water supplies and coastal marine fisheries.

Errors in mass balance calculations like those described above most likely arise from over- or underestimates of the rates of specific steps in the N cycle. For example, the "missing" N may result from poor or absent information on plant uptake and storage, immobilization and denitrification in soils, loss during passage through wetlands and riparian zones, and uptake and denitrification in lakes, streams, and large rivers. Historically, ecologists have considered lotic habitats to be passive conduits or subcompartments of limited importance found within the integrated watershed ecosystem[16,17,18]. The presumption that streams are passive with respect to N export may have derived from the observation that much of the inorganic N transported by streams and rivers is in the form of $NO_3$-N, a highly mobile N species thought to move rapidly downstream towards lakes and coastal ecosystems.

In a recent mass balance analysis of the Mississippi River, Alexander et al.[19] illustrated that a large percentage of total N and $NO_3$-N were lost as water traveled through low-order tributary streams, drawing attention to the role of streams in N processing and questioning previously held views regarding stream N processes. Loss rates within stream channels were as high as 50% $day^{-1}$ in streams with depths <50 cm, but declined rapidly to ~0.5% $day^{-1}$ in rivers several meters deep. Thus, Alexander et al. concluded that only a fraction of lotic N escaped small streams (lower than fourth-order) but that N reaching large river channels traveled readily to the mouth of the Mississippi[19]. These findings strongly suggest that stream ecosystems themselves, especially headwater streams, are important sinks for anthropogenic N. Our current understanding of N cycling and transport in streams quantifies these processes using the concept of nutrient spiraling[20,21]. Below, we discuss how a spiraling approach can be used to understand how headwater streams act as sinks for N and what features of low-order streams cause them to act in this manner.

We have learned much from decades of research on various aspects of N dynamics in stream channels, but most of that research has been carried out in piecemeal fashion and a comprehensive synthesis is just now becoming available. The working hypotheses of this paper are that headwater streams are critical points of N retention across the landscape that are characterized by efficient spiraling and retention because: (1) much of the stream length within a catchment is located in its headwaters where land-water interface is maximal; (2) headwater streams exhibit high surface:volume ratios that enhance the influence of biotic activity on water chemistry; and (3) low-order streams may include a subsurface component that is metabolically active, biogeochemically retentive, and hydrologically linked to the surface stream. Here, we provide a brief review of N cycling in streams and present literature values of cycling rate constants and spiraling indices. We then discuss how hydrologic linkage of surface and subsurface environments influences nitrogen cycling and present a conceptual model of N dynamics for headwater streams. We end by developing a series of question sets and accompanying hypotheses we hope stimulates future research.

## N CYCLING AND SPIRALING IN STREAMS

While streams and rivers drain catchments and act as large-scale transport systems, a number of processes within lotic ecosystems retain and transform materials that would otherwise move downstream[22,23,24,25,26,27]. Though removal and regeneration of specific nutrients may be considerable, little net effect may be observed in solute concentrations if these activities are in balance. In fact, streams have little ability to influence the long-term transport of elements such as phosphorus that have no gaseous phase that readily exchanges with the atmosphere[28]. However, even in these cases biotic activities within streams alter the chemical form of nutrients and modify the timing of their transport. In contrast, elements that have gaseous phases, such as carbon (C) and N, may be lost from the system to the atmosphere, and ecological processing may significantly alter the long-term transport of these elements downstream. As with the processing of all materials in lotic ecosystems, N dynamics are influenced by downstream transport that results in "open" cycles referred to as nutrient spirals[20,29,30].

Nutrient cycling involves the exchange of specific elements between ecosystem components, and the associated transformations that occur during these processes (Fig. 1). Conceptually, cycling occurs at any single location within a stream and can be quantified on either an areal or volumetric basis (e.g., assimilatory uptake rates in mg $m^{-2}$ $day^{-1}$) despite the fact that individual atoms never complete an entire cycle at any one location. Spiraling, in contrast, addresses both cycling and downstream transport and seeks to quantify the longitudinal distances over which complete cycles occur (spiral length) or those distances associated with specific cycling steps (uptake length, turnover length, and seston and dissolved organic carbon [DOC] transport distances; Fig. 2). More information regarding the derivation and relationships among spiraling parameters may be found in existing reviews that provide in-depth discussions of nutrient dynamics and spiraling in streams[28,31,32].

The full complement of N transformations occurs in stream ecosystems, including N fixation, nitrification, denitrification, assimilation (of ammonia and nitrate), and ammonification (Table 1). As indicated above, N cycling also can be expressed in a spatial context using indices such as uptake length and uptake velocity (Table 1) or turnover length and spiral length. Availability of relatively inexpensive stable isotopes ($^{15}N$-$NH_4$ and $^{15}N$-$NO_3$) has led to several field tracer experiments aimed at quantifying many of these processes and providing an initial assessment of factors influencing N-spiraling rates and distances[33,34].

## THE FUNCTIONAL SIGNIFICANCE OF THE HYPORHEIC ZONE

Historically, streams and rivers have been viewed as two-component ecosystems composed of the stream bottom (benthic zone) and overlying water column. However, since the eloquent pleas

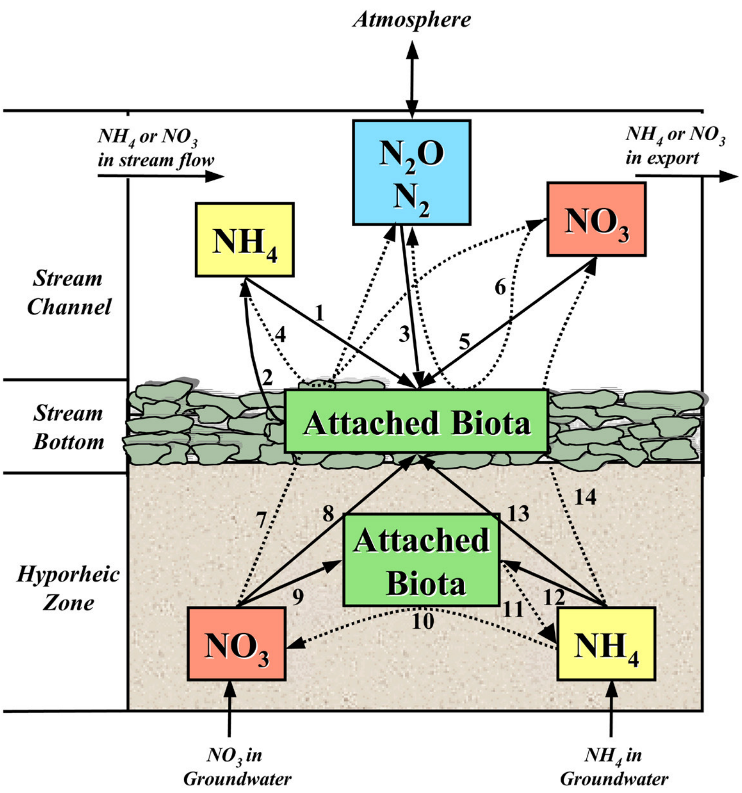

| Pathway | Process | Selected References |
|---|---|---|
| 1 | $NH_4$ assimilation | 25,33,34,78,79 |
| 2 | Ammonification | 33,34,78 |
| 3 | N fixation | 77 |
| 4 | Nitrification | 34,78,79,86,87 |
| 5 | Assim. $NO_3$ Reduction | 27,34,83,85,86 |
| 6 | Denitrification | 80,81,82,88 |
| 7 | Denitrification | 45,46,88 |
| 8 | Assim. $NO_3$ Reduction | 47 |
| 9 | Assim. $NO_3$ Reduction | 88 |
| 10 | Nitrification | 41,61,88,89 |
| 11 | Ammonification | 41,89 |
| 12 | $NH_4$ assimilation | 90 |
| 13 | $NH_4$ assimilation | 90 |
| 14 | Nitrification | 88,90 |

**FIGURE 1.** Conceptual model of N dynamics in streams. In this representation, an upwelling zone (scale = meters) is depicted. Though not represented in this figure, channel and hyporheic pools of $NO_3$ and $NH_4$ are hydrologically linked. Solid and dashed lines represent assimilatory and dissimilatory pathways, respectively. Dissimilatory nitrate reduction (DNR) is not in this figure because it is generally assumed to be small in streams, though our level of understanding of DNR is very low.

of Hynes, Danielopol, and Ward, stream ecologists have expanded the physical and conceptual boundaries of lotic ecosystems to include closely associated groundwater environments that interact with the benthic zone and water column (i.e., hyporheic zones)[35,36,37,38,39]. The hyporheic zone is metabolically active and may significantly influence a stream's organic matter budget[40,41,42,43]. In addition, physical-chemical conditions within hyporheic zones may support a suite of biogeochemical processes that rarely occur on the surface, including denitrification[4,44,45,46]. Finally, because hyporheic zones exchange flow with the surface stream, processes within them may strongly influence nutrient form and abundance in both stream and groundwater ecosystems and significantly influence the spatial and temporal heterogeneity of biotic processes on the stream benthos[47,48,49,50,51,52].

The extent of groundwater-surface water (GW-SW) exchange and the size of the hyporheic zone in streams vary in space and time. Among streams, differences in the size of the hyporheic zone reflect variations in geologic composition and sediment volume[27,48,52,53,54]. Within a stream, the size of the hyporheic zone may vary with reach geomorphology[55,56]. Fernald et al.[57] measured larger transient storage zones (a hydrologic parameter used to represent hyporheic zone size) in unconstrained reaches of the eighth-order Willamette River than in narrower reaches with less alluvial storage. D'Angelo et al.[55] observed a similar trend for montane headwater streams in Oregon. Research on headwater streams in New Mexico has shown that hyporheic zone size within a single stream also varies with time. Variation in discharge over the course of summer baseflow and spring snow melt generated a 460-fold change in the size of the hyporheic zone relative to the stream channel[54]. Together, these studies show that hyporheic zone size relates to structural characteristics of the stream channel and that the extent of interaction is temporally variable in response to changing flow conditions.

Currently, views differ on the relative importance of subsurface processes to the functioning of stream ecosystems. Some studies have shown that the entire volume of surface water is cycled through the hyporheic zone in <100 m of stream channel[52,54]. Other studies have indicated that in comparison with surface fluxes and processing rates, hyporheic influences are quantitatively insignificant[58,59]. Recent studies suggest that nutrient retention is closely related to GW-SW exchange as evidenced by significant declines in uptake lengths for $NO_3$-N and phosphate-phosphorus ($PO_4$-P) with increasing hyporheic size[27,48,53]. In addition, investigations of stream metabolism and GW-SW interaction support the view that hyporheic zones contribute significantly to energy flow in streams[48,60]. Because hyporheic metabolic processes are predominantly heterotrophic, larger hyporheic zones appear to increase whole-system respiration[40,43,48,60,61,62].

Respiration in groundwater ecosystems, including hyporheic zones, is limited by carbon availability[41,61,63,64,65]. Sources of organic carbon differ greatly among streams[66]. Thus, variation in hyporheic metabolism may be the result of variation

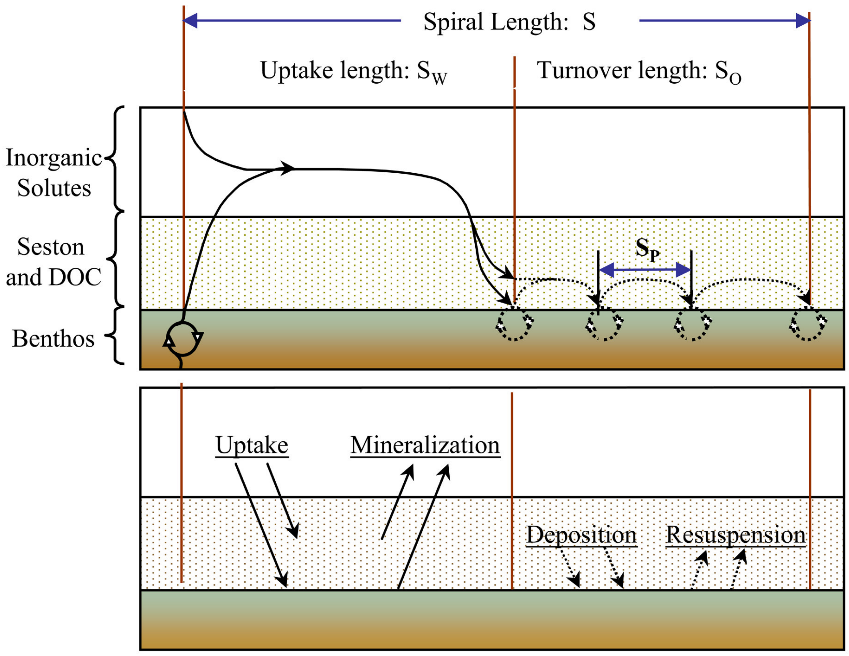

**FIGURE 2.** Nutrient spiraling in stream ecosystems. Solid arrows represent travel in an inorganic form while dashed lines represent transport in organic forms. The small cycles in the benthos represent the potential for cycling activity to occur within streambed sediments at an individual site (e.g., within a periphytic biofilm). $S_W$ = uptake length, $S_O$ = turnover length, and $S_P$ = particle transport distance. Seston is suspended, particulate organic matter consisting primarily of particles <250 µm in diameter. Dissolved organic carbon (DOC) consists of a multitude of organic compounds <0.45 µm in diameter. Uptake and mineralization refer to the primary mechanisms through which atoms travel between inorganic and organic forms, respectively. Similarly, deposition and suspension describe the flux of particulate organic carbon (POC) between the bed and the water column. The fluxes of DOC into and out of POC (not represented in this figure) are referred to as assimilation and excretion. (Modified from Newbold[28].)

## TABLE 1
## N Transformation Rates and Spiraling Indices for Selected Headwater Streams, U.S., North America

| N Flux | Site | Transformation Rate (mg N $m^{-2}$ $d^{-1}$) | Reference |
|---|---|---|---|
| N - Fixation | desert stream, AZ | 42 - 144 | 77 |
| nitrification | temperate woodland stream, TN | 6 | 33 |
| | prairie stream, KN | 0.2 - 2.6 | 78 |
| | woodland stream, NC | 6 | 79 |
| denitrification | stream periphyton chambers, CA | 18 | 80 |
| | deciduous forest streams, TN | 0.5 | 81 |
| | acid/N -depositional forested stream, TN | 6.8 | 81 |
| | N-enriched stream sediments, IN | 320 | 82 |
| N uptake | desert stream, AZ | 250 | 25 |
| | coniferous forests streams, NM | 3 - 107 | 27 |
| | coniferous forest stream, OR | 7 | 83 |

| Spiraling Index | Site | Index Value | Reference |
|---|---|---|---|
| uptake length (m) | coniferous forest stream, OR | 17 | 83 |
| | desert stream, AZ | 60 - 190 | 84 |
| | coniferous forests streams, NM | 133 - 3183 | 27 |
| | montane coniferous forest streams, ID | 549 - 1839 | 85 |
| uptake velocity (mm/s) | coniferous forest stream, OR | 0.041 | 83 |
| | desert stream, AZ | 0.018 - 0.16 | 84 |
| | coniferous forests streams, NM | 0.006 - 0.010 | 27 |
| | montane coniferous forest streams, ID | 0.02 - 0.08 | 85 |

in the quantity and quality of organic matter inputs to streams. For instance, litter inputs to Walker Branch, TN and Hugh White Creek, NC represented 88 and 98% of annual organic carbon inputs, while litter inputs to streams in Arizona and New Mexico are less than 5% of annual organic carbon production[48,67,68,69]. While Jones et al.[41] clearly demonstrated that hyporheic metabolism in Sycamore Creek, AZ was tightly coupled to import of dissolved organic carbon from the surface stream, little is known about how natural variation in organic carbon availability influences hyporheic metabolism and N cycling.

Extended water residence times along metabolically active hyporheic flow paths may generate anoxia and support anaerobic processes despite low ambient organic carbon concentrations[4,27,44]. Valett et al.[27] demonstrated that $NO_3$-N retention increased with decreasing hyporheic oxygen content, suggesting an important role for denitrification. Other studies that have quantified hyporheic denitrification in headwater streams also have shown its importance even when extensive zones of anoxia are absent in stream sediments[46,70]. Baker et al.[65] and Morrice et al.[71] quantified respiration and consumption of terminal-electron acceptors in the hyporheic zone of Rio Calaveras, NM and found that anaerobic processes dominated hyporheic metabolism. Over 90% of injected $NO_3$-N was removed along the first 30 cm of a hyporheic flow path[71]. Baker et al.[72] showed that injection of acetate (a labile carbon source) along the same flow paths increased respiration rates and enhanced $NO_3$-N consumption.

Recently, Mulholland et al.[48] developed a means of segregating hyporheic and surface retention of $^{33}PO_4$-P using whole-stream injection experiments. In their research, an analysis of the temporal profiles of the ratio of the reactive tracer to the conservative tracer was used to examine the contribution of surface process to overall P retention. Their analysis used [$^{33}PO_4$-P]:[conservative tracer] ratios during the initial stages of the rising limb of the tracer breakthrough curve to delineate the effect of surface uptake processes and ratios measured during the steady-state portion of the experiment to estimate surface plus hyporheic processes. The proportion of $PO_4$-P retained by hyporheic processes was determined by difference. We are currently employing similar methods with field additions of $^{15}N$-$NO_3$ and have estimated that subsurface contribution to $NO_3$-N retention frequently may be more than 50% of total uptake (unpublished data).

## A CONCEPTUAL MODEL FOR NUTRIENT RETENTION IN STREAM ECOSYSTEMS

Our studies of nutrient dynamics and GW-SW exchange have generated a conceptual model of retention that emphasizes both the hydrology of GW-SW interactions and rates of biological and chemical processes[27,53,58]. Valett et al.[27] presented ecosystem retention as the product of water residence time (i.e., hydrologic retention) and the rates of processes sequestering or transforming nutrients. Greater GW-SW interaction increases hydrologic retention and enhances the potential for nutrient retention. In Fig. 3, we expand the original model to emphasize the

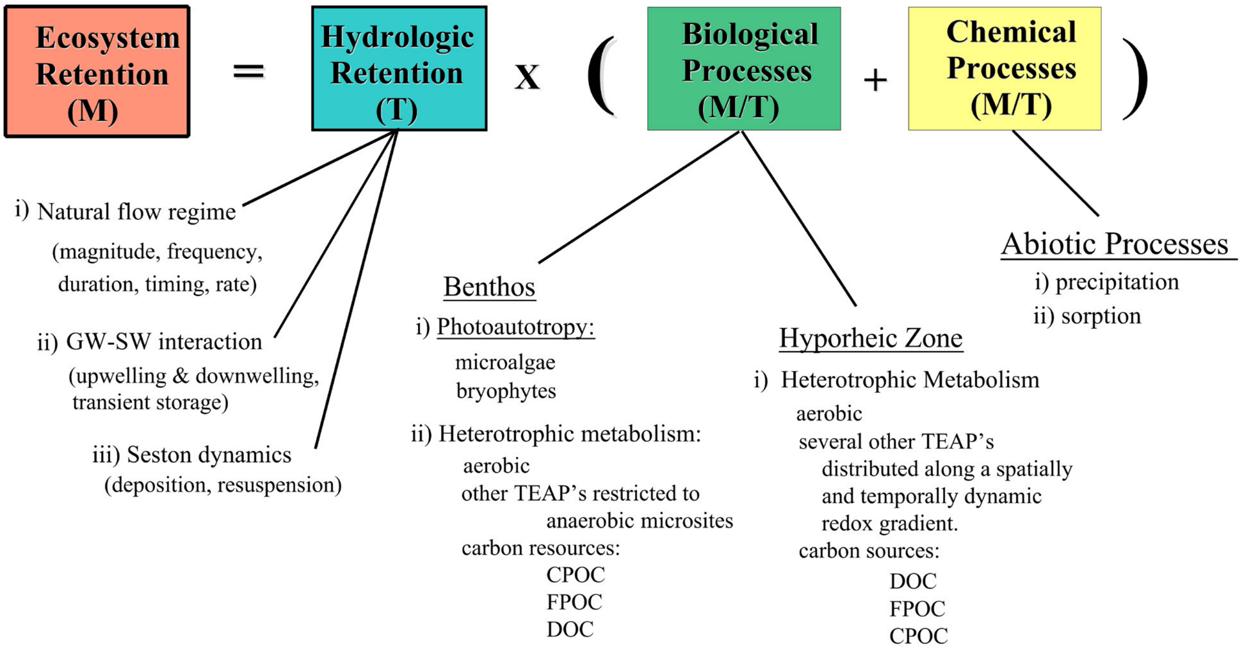

**FIGURE 3.** Expanded model of nutrient retention. Hydrologic retention denotes increased water residence time that results from GW-SW interaction, the magnitude of which may vary among catchments and over the course of the natural flow regime. Ecosystem retention is the product of hydrologic retention and rates of biological or chemical processes that may occur within the channel, benthic, or hyporheic subsystems. Benthic compartments actively involved in retention include periphytic biofilms and microbial communities associated with various organic matter pools. Processes active in retaining/transforming nutrients in the benthic zone include photoautotropy, aerobic microbial production, and various terminal electron–accepting processes (TEAPs) occurring in anaerobic microsites. Retentive activities in the hyporheic zone are predominantly heterotrophic and include the suite of TEAPs distributed across a spatially complex redox gradient. Organic carbon (OC) sources are listed in the order of relative importance. The abiotic processes of precipitation and sorption may sequester nutrients from flow in surface and subsurface compartments. (After Valett et al.[27].) M and T indicate units of mass and time respectively.

mechanisms responsible for biological uptake and total ecosystem retention of nutrients.

While hydrogeologic properties of stream sediments set broad controls on GW-SW interaction, hydrologic retention will also vary temporally with seasonal or episodic fluctuation of the natural flow regime within any given stream[54,73]. Understanding the importance of subsurface processes to nutrient retention requires considering how their contribution varies between periods of low and elevated discharge. Four principle organic matter compartments contribute to surface metabolism and nutrient retention: (1) autotrophs (e.g., benthic algal communities, bryophytes), (2) coarse particulate organic matter (CPOM, mainly leaves and woody debris), (3) fine particulate organic matter (FPOM, smaller-sized organic particles), and (4) DOC. Macroinvertebrate feeding, microbial assimilation, leaching, and physical abrasion mediate the transfer of material among these. Standing stocks of organic matter in these pools vary among streams and with season and discharge. Variations in these aspects of stream structure reflect differences in canopy development (through the effects on light availability and the magnitude of allochthonous inputs) and organic matter processing by grazing and detritus-feeding macroinvertebrates. Microbial activity associated with each of these organic matter pools has great potential to influence nutrient retention, as may the transport dynamics of FPOM[74].

Subsurface processes use solutes through assimilatory and dissimilatory pathways. After the consumption of dissolved oxygen through aerobic respiration, a series of terminal electron–accepting processes (TEAPs) consume and transform various solutes during anaerobic metabolism. In the absence of organic contamination, rates of subsurface TEAPs are generally limited by the availability of labile organic carbon substrates[75]. Relatively few studies, however, have addressed how natural variations in rates of subsurface metabolic and biogeochemical processes influence the transport and retention of nutrients by stream ecosystems.

## CURRENT AND FUTURE RESEARCH DIRECTIONS

Because $NO_3$-N is a highly mobile form of N and does not adsorb significantly to stream sediments, retention results almost exclusively from the biotic processes of assimilation and transformation[26,76]. Nevertheless, very little work has investigated the link between metabolic rates and nutrient retention, and investigations of these relationships in hyporheic environments are limited to those discussed above. In an attempt to stimulate research in this area, we present several research questions and testable hypotheses as potentially fruitful areas of research that may lead to better understanding of the types and rates of stream processes impacting N transformation and retention.

### Question Set 1

How do interactions between streams and their hyporheic zones influence metabolism and $NO_3$-N retention? How does the importance of subsurface $NO_3$-N retention vary among streams of different regions and within streams as discharge varies?

#### *Testable hypotheses:*

1. Among streams, $NO_3$-N uptake lengths and subsurface uptake rates will correlate with the level of interaction between the stream and the storage zone.
2. Subsurface $NO_3$-N uptake rates will correlate with hyporheic respiration rates.
3. Percent total retention due to subsurface uptake will be inversely related to benthic organic matter standing stocks.
4. Subsurface $NO_3$-N uptake rates will be greatest during low flow when surface-subsurface exchange is maximal.

### Question Set 2

How do different metabolic processes (i.e., primary production vs. heterotrophic organic matter utilization) influence $NO_3$-N retention in lotic ecosystems? How does their relative importance vary spatially across the landscape and temporally within streams?

#### *Testable hypotheses:*

1. Streams with greater metabolic activity will have higher rates of $NO_3$-N retention, and uptake rates will correlate with metabolic rate (gross primary production, respiration).
2. Night and day uptake lengths in streams of well-shaded catchments will be more similar than those in open-canopied systems where growth of photoautotrophs is a significant daytime $NO_3$-N sink.
3. Within streams with large seasonality in allochthonous and autochthonous carbon inputs (e.g., Walker Branch), day and night uptake lengths should be most similar following leaf fall and most dissimilar in times of maximum insulation (early spring).

### Question Set 3

How do rates of denitrification vary among headwater streams in regions with contrasting organic carbon inputs? How do changes in carbon availability alter rates of denitrification? How does GW-SW exchange affect denitrification rates?

#### *Testable hypotheses:*

1. Denitrification rates will be greatest in streams with higher availability of organic carbon and $NO_3$-N.
2. Rates of denitrification will be greatest during times of increased carbon availability (i.e., fall leaf litter inputs in canopied streams) and enhanced subsurface anoxia (summer baseflow in open systems).
3. Experimentally increasing labile organic carbon availability will increase denitrification rates.
4. Denitrification rates will be greatest under conditions where GW-SW exchange promotes regions of low redox potential.

## CONCLUSIONS

Alexander et al.[19] indicated that the vast majority of N retention in lotic networks occurs in the headwaters. The implications of this study are of practical significance because they suggest a strategy for controlling coastal zone pollution — routing N through a series of small streams and rivers where maximal N loss will occur. This conclusion is consistent with results from a recently completed study of ammonium-N ($NH_4$–N) dynamics in streams throughout North America (the Lotic Intersite Nitrogen Experiment, LINX)[34]. This intersite $^{15}$N-tracer study demonstrated experimentally that smaller streams are most retentive of $NH_4$-N, with shortest ammonium uptake distances (i.e., greatest retention efficiencies) in the smallest streams and uptake distances increasing logarithmically with stream discharge and depth. In headwater streams throughout North America, Peterson et al.[34] found that less than 50% of the $NH_4$-N entering a stream remained in transport after traveling 1 km downstream.

We suggest that one reason for the highly retentive character of these small streams is the extensive interaction between surface and subsurface (hyporheic) environments. We also suggest that this interaction facilitates retention by providing a gradient of redox conditions that sustain various N-transforming processes. Increasing availability of fixed N constitutes one of the most obvious global changes induced by humans. Coupling this observation with growing concerns over drinking-water supplies and other aquatic ecosystem services leads us to believe that interest will continue to grow regarding the remediation abilities of lotic habitats. Hopefully, this presentation has portrayed headwater streams as keystone ecosystems with respect to N dynamics.

| | | |
|---|---|---|
| Received: | August | 8, 2001 |
| Revised: | September | 25, 2001 |
| Accepted: | October | 5, 2001 |
| Published: | November | 9, 2001 |
